# Preliminary feasibility study on DTI to assess the early brain injury in germinal matrix-intraventricular hemorrhage rats

**DOI:** 10.1038/s41598-025-94934-x

**Published:** 2025-03-21

**Authors:** Chi Qin, Chenxi Guo, Huixian Li, Ronghao Mu, Meiying Cheng, Haiyang Li, Xiang Feng, Bohao Zhang, Yue Li, Jian Jin, Xin Zhao, Xiaoan Zhang

**Affiliations:** 1 Department of Radiology, The Third Affiliated Hospital of Zhengzhou University, Zhengzhou, China; 2 Department of Clinical Research and Translational Medicine, The Third Affiliated Hospital of Zhengzhou University, Zhengzhou, China; 3 Department of Child Developmental Behavior, The Third Affiliated Hospital of Zhengzhou University, Zhengzhou, China; 4 MRSolutions Ltd., Guildford, Surrey GU3 1LR UK; 5 Sydney Imaging, Core Research Facility, The University of Sydney, Sydney, NSW 2006 Australia

**Keywords:** Diffusion tensor image, Germinal matrix-intraventricular hemorrhage, Pathological changes, Motor and cognitive impairment, Neuroscience, Development of the nervous system, Diseases of the nervous system, Learning and memory

## Abstract

To evaluate the feasibility and efficacy of diffusion tensor imaging (DTI) for detecting early brain microstructure alterations in germinal matrix-intraventricular hemorrhage (GMH-IVH) rat model. This study used a postnatal day 5 (PND 5) rat model of GMH-IVH. T2-weighted imaging and DTI were performed during acute (6 h and 24 h) and subacute (3d and 7d) phases after GMH-IVH. Four DTI parameters including fractional anisotropy (FA), mean diffusion (MD), axial diffusion (AD) and radial diffusion (RD) were collected in 9 specific brain regions to assess the brain microstructure alterations. Early and long-term neurological function tests were evaluated. Transcriptome sequencing analysis was also performed to investigate possible underlying mechanisms. Regional abnormalities after GMH-IVH were observed in T2-weighted images that showed significant hypointense in striatum region which close to the germinal matrix. DTI parameters also observed changes in striatum region in GMH-IVH. Alterations in other regions of brain including hippocampus, thalamus, external capsule and motor cortex also noted, which were associated with the abnormalities observed in behavioral experiments. Long-term behavioral tests show that compared to sham group, rats in GMH-IVH group caused abnormal motor function. In addition, at 24 h after GMH-IVH, transcriptome analysis results showed that the highly expressed differential genes encode hemoglobin components and down-regulate neurodevelopment-related pathways. DTI imaging allows the early assessment of neurological alteration in GMH-IVH rat pups, and providing great value in evaluating long-term behavioral deficits.

**Supplementary Information:**

The online version contains supplementary material available at 10.1038/s41598-025-94934-x.

## Introduction

Preterm birth is considered a global burden and is the leading cause of neonatal death. In the last 10 years, approximately 15% of all preterm births were born at less than 32 weeks of gestation^1^. Germinal matrix-intraventricular hemorrhage (GMH-IVH) is the common neurologic complication in preterm neonates, which is associated with low birth weight (< 1500 g) and/or preterm birth (< 32 weeks gestation)^2–4^. The incidence is approximately 20–25% of GMH-IVH in neonates with very low birthweight^5,6^. GM-IVH is categorized into four grades according to Papile grading system^7^. Grade I and II are considered as mild GMH-IVH, grades III and IV are known as severe GMH-IVH. GMH-IVH still to be the major complications of prematurity that result in white matter injury (WMI)^8^ and long-term negative neurodevelopmental outcomes in survivors^9^. Currently, therapies for GMH-IVH neonates are limited to rehabilitated care and potential treatment are undergoing investigation in preclinical models^2,8,10^.

The pathogenesis and mechanism of GMH-IVH are multifactorial and not well understood^2,11^. Several preclinical models show that a series of pathological reactions, including oxidative stress, glutamate excitotoxicity, neuroinflammation and signaling pathway disturbance es can induce white matter injury (WMI)^8,12^. Myelin basic protein (MBP) is usually used as an objective biomarker of central nervous system damage and acute demyelination which reflect the severity of myelin damage of white matter oligodendrocytes^13,14^. However, MBP staining is mainly presented by immunofluorescence technique and is difficult to dynamically monitor and evaluate changes of white matter microstructural in real time. Therefore, it is necessary to explore an effective non-invasive biomarker.

Diffusion tensor imaging (DTI) is a non-invasive advanced MRI technique that allows for in vivo assessment of the restricted movements of water molecules in white matter and provided microstructural damages. Fractional anisotropy (FA) and mean diffusivity (MD) was the frequently used quantitative DTI parameters. While, FA refers to the anisotropy of diffusion, MD reflects the mean magnitude of diffusion. In principle, the directional diffusivity modulated by the microscopic movement of water molecules has been evidenced to be parallel (axial diffusivity, AD) and perpendicular (radial diffusivity, RD). Recently, it has been applied in many animal models including global cerebral ischemia/reperfusion injury^15^ and hypoxic-ischemic encephalopathy^16^. In 2022, Zhang et al.^17^ first used DTI to evaluate the different brain regions after germinal matrix hemorrhage. They found the thalamus far from the hematoma also was damaged. However, they performed DTI scanning only at 40 days after germinal matrix hemorrhage and ignored changes of DTI parameters during the acute and subacute phase. Previous study performed the temporal transcriptome profile and provided a comprehensive understanding of mechanism of brain injury after germinal matrix hemorrhage in neonatal rats. Therefore, in this study, we tested for DTI parameters (including FA, MD, AD and RD) at 6 h, 24 h, 3days and 7days in rats after GMH-IVH. We compared these parameters with histological results and locomotor outcomes and examined their consistency and capability of reflecting spatial white matter in time. In addition, the RNA sequencing was performed to further explore the mechanism of brain injury in early stage caused by GMH-IVH.

The purpose of this study was to evaluate the early dynamic changes in MRI parameters and to evaluate neurobehavioral changes in neonatal rat GMH-IVH model, providing a basis for non-invasive monitoring GMH-IVH induced brain injury.

## Methods

### Animals

Sprague-Dawley (SD) rats were used from the Laboratory Animal Center [ Henan, China]. All animals were housed under conditions of the ambient temperature of 22 ± 2 ℃, a humidity of 56 ± 5%, and 12 h light/dark cycle. One hundred and thirty PND 5 SD neonatal pups were randomly divided into sham and GMH-IVH groups (Supplementary Table 1). The inclusion criteria were the pups with both genders and the body weight of 9–12 g at PND 5. The exclusion criteria were as follows: (1) animals died during the experiments and (2) rats without hemorrhage after GMH-IVH induction. All experimental procedures were approved by the Institutional Animal Care and Use Committee of The Third affiliated hospital of Zhengzhou University (ethical number: 2023-300-01). To minimize pain or stress in rats, animals’ euthanasia process in this study by using CO_2_ inhalation. Euthanasia was performed by using an inhouse-designed euthanasia system at volume displacement rate per minute of 10%.

### Germinal matrix-intraventricular hemorrhage (GMH-IVH) model

The GMH-IVH model was performed in unsexed PND 5 rats using collagenase infusion. Simply, pups were anesthetized with isoflurane (3.5% for induction and 1.5-2% for maintenance). The scalp was incised along the longitudinal plane and gently exposing the bregma, then a 1-mm burr hole was drilled into the skull. A 28-gauge needle with 0.3 U collagenase VII (Sigma) in 2 ml saline or saline alone was injected into the right middle medial striatum for induction of GMH-IVH or sham group as previously described^18^.

The injection site was the right hemisphere: 1 mm rostral of the bregma, 3 mm lateral of the midline and 4.0 mm in depth through the burr hole and infused at 1 µl/min for 2 min using a 25 µl Hamilton syringe. After infusion, the syringe remained at the site for 6 min to avoid backflow and withdrawn at speed of 0.5 mm/min. Skin covering the burr hole was sutured closed following procedure and the pups were placed onto a 35 ℃ heated blanker for recovery.

### Magnetic resonance imaging (MRI) acquisition

All imaging was performed on a 4.7T horizontal cryogen-free preclinical MR system (MR Solutions, Guildford, Surrey, United Kingdom). A quadrature rat brain coil was used. During the MRI acquisition, rats were anesthetized using isoflurane (3.5% for induction and 1.5% for maintenance). For anatomical scans, T2-weighted Fast Spin Echo images were acquired: 20 slices, slice thickness = 1 mm, no slice gap, TR = 4000 ms, TE = 51ms, echo train = 7, average = 3, matrix = 256 × 238, FOV = 25 × 25 mm², and total scan time = 7 min.

To evaluate the white matter injury, the DTI images were acquired with a 2-shot spin-echo EPI sequence with one navigator and read interleave mode: 1b0 and 66 directions of b = 1000s/ mm², 20 slices, slice thickness = 1 mm, no slice gap, in-plane spatial resolution 0.172 × 0.172 mm^2^, TR = 5000 ms, TE = 27 ms, δ = 5 ms, △= 13 ms, average = 1, matrix = 100 × 70 (partial Fourier along Phase encoding direction), reconstructed matrix after interpolation = 128 × 128, receiver bandwidth = 200 kHz, and total scan time = 22 min, 40 s.

### Regions of interests and image processing

Two observers (Chenxi Guo and Xin Zhao) manually draw nine brain regions on the DWI images including striatum, corpus callosum, external capsule, internal capsule, hippocampus, hypothalamus, motor cortex, somatosensory cortex, and thalamus as the regions of interests for analysis. The selected ROIs was determined at the level where the nine brain regions exhibited the clearest visualization, with bilateral measurements taken. To minimize manual drawing errors, the regions of interest (ROIs) were saved as templates for labeling. DTI metrics (FA, MD, AD, and RD) of the white matter were calculated using DSI studio (http://dsi-studio.labsolver.org) software for each rat and at each time point. To facilitate visual inspection of the image, the brain tissue mask was applied. The 3D visualization of fiber tracts in brain white matter were generated using a deterministic fiber tracking algorithm. The interclass correlation coefficients (ICCs) was used to evaluate the consistency test between two operators. Additionally, each operator repeated the measurements three times, and an intra-group consistency test was performed.

### Evaluation of early development of neonatal rats

To better reflect the development of neonatal rat pups after collagenase injection, we collected pups body weight and weight gain rate (WGR) over a period of 7 days. The formula of WGR is: WGR = (Final weight – Original weight)/original weight × 100%. Negative geotaxis test is used to examine the time required for a pup to rotate 180° after placing the head down at a 20° slope.

### Long-term behavior tests

For all behavior tests, we conducted two batches of behavior tests, first in PND 35–42, and the second in PND 65–72.

### Rotarod test

The rotarod test was used to assess movement and balance in rats on PND 35 and PND 65. Before the start of the experiment, rats underwent gradient training of 15r/min to 30r/min 3 days in advance for 15 min every day. In the formal test, the adjustment speed was 30r/min, and the latency to fall was measured. If the mouse did not drop within 5 min, its time on the rod was recorded as 5 min. And the apparatus was cleaned with 75% alcohol purge between each test.

### Open field test (OFT)

At PND 36 and PND 56, locomotor activity was evaluated in a black acrylic which also named an open field chamber (100 cm × 100 cm × 40 cm). During the procedure, each rat was placed in a corner of the chamber and allowed to move freely for 10 min. The locomotion parameters such as the total distance, the time spent in center and the average speed in center were recorded.

### Y-maze

As described previously, to evaluate spatial working memory of spontaneous alteration, Y-maze (three arms, 100 cm long × 20 cm wide × 40 cm high) were conducted. Rats were placed in the intersection of three arms to choose any arm, and all results were recorded during a 5 min exploration experiment by camera. Before trial, all rats were proper trained to familiar with the maze. The percentage of spontaneous alteration was defined according to the following calculation: (numbers of spontaneous alteration) / (total number of arm visits – 2).

### Gait analysis

Gait and postural parameters were analyzed at PND 42 or PND 72 using the CatWalk XT system (Noldus Information Technology, Wageningen, the Netherlands). The CatWalk XT system consisted of a 1.3-m-long horizontal glass panel which covered by a removable tunnel creating a dimmed light on the walkway. A green LED light is emitted inside the glass plate and could reflect in computer. Rat pups were placed at the beginning of the pathway and willing to traverse the plate to their cage. When their paws touch the glass plate, light is refracted on the opposite side. And all of these illuminated areas are automatically recognized by a high-speed color camera which is located underneath the glass plate. Some parameters were set before the experiment, including a maximum speed variation of rat pups was 60%, a camera gain of 23.4 dB, and the detection threshold was 0.12. After 5 consecutive and successive training days, these rat pups underwent at least three uninterrupted runs to qualify for CatWalk XT analysis on test day.

### Brain histology

Rat pups were sacrificed for brain histological assessment. Corresponding to the T2WI scanning location, tissues containing the anatomical layer to observe were cut for freezing embedding as previously displayed. Briefly, animals were anaesthetized and trans-cardiac perfusion with phosphate buffered saline (PBS). After an ice-cold 4% paraformaldehyde (PFA) perfusion, brain was removed and fixed in formalin overnight at 4℃. Brain samples were sectioned into 4-µm-thick coronal segments using a paraffin microtome (Leica, Germany). Brain tissue sections were stained with hematoxylin-eosin (H&E).

### RNA-sequencing and analysis

Total RNA was extracted from the injury brain tissue of GMH-IVH and the same location of control group using TRIzon Reagent (CW0580S, CWBIO, China) according to the manufacturer’s instructions. RNA quantified using a spectrophotometer (NanoDrop 2000, Thermo Scientific) at 260 nm absorbance. Total RNA was extracted from five sample replicates in GMH-IVH group and control group. Sequencing libraries were generated by using the NEBNext Ultra Directional RNA Library Prep Kit for Illumina (New England Biolabs) and generating 150 bp paired-end reads based on the manufacturer’s instructions. Gene expression levels and differentially expressed genes (DEGs) were computes according to DEGseq R package. Based on DEGs, gene ontology (GO) and kyoto encyclopedia of genes and genomes (KEGG) pathway enrichment analysis were further performed. GO enrichment analysis of DEGs was implemented by the clusterProfiler packages (https://bioconductor.org/packages/release/bioc/html/clusterProfiler.html) based Wallenius non-central hyper-geometric distribution, which can adjust for gene length bias in DEGs. KEGG^19^ is a large-scale molecular dataset generated by genome sequencing and other high-throughput experimental technologies (www.kegg.jp/kegg/kegg1.html). We used KOBAS database and clusterProfiler software to test the statistical enrichment of differential expression genes in KEGG pathways. All the analysis was performed on BMKCloud (www.biocloud.net ).

### Statistical analysis

All statistical analyses were used GraphPad Prism (CA, USA) and SPSS 21.0 (IBM, USA). Sample size in this study was based on the previous reports^17,20^. All the data were first tested for normal distribution using Shapiro-Wilk test, and then further statistical analysis was performed. Quantitative data are represented as mean ± standard deviation, while qualitative data are presented as frequency and percentage. Intergroup differences among DTI parameters were evaluated using two-way repeated measure analysis of variance (ANOVA). Post-hoc multiple comparison analysis using Tukey corrections. The intraclass correlation coefficient (ICC) was calculated to evaluate agreement for DTI measurements. A two-tailed unpaired t-test or Mann-Whitney U-test was used to compare the sham and GMH-IVH groups in neurobehavior tests. *P* < 0.05 was considered statistically significant.

## Results

### Alterations in brain DTI parameters after GMH-IVH rat pups

**Fig. 1:**
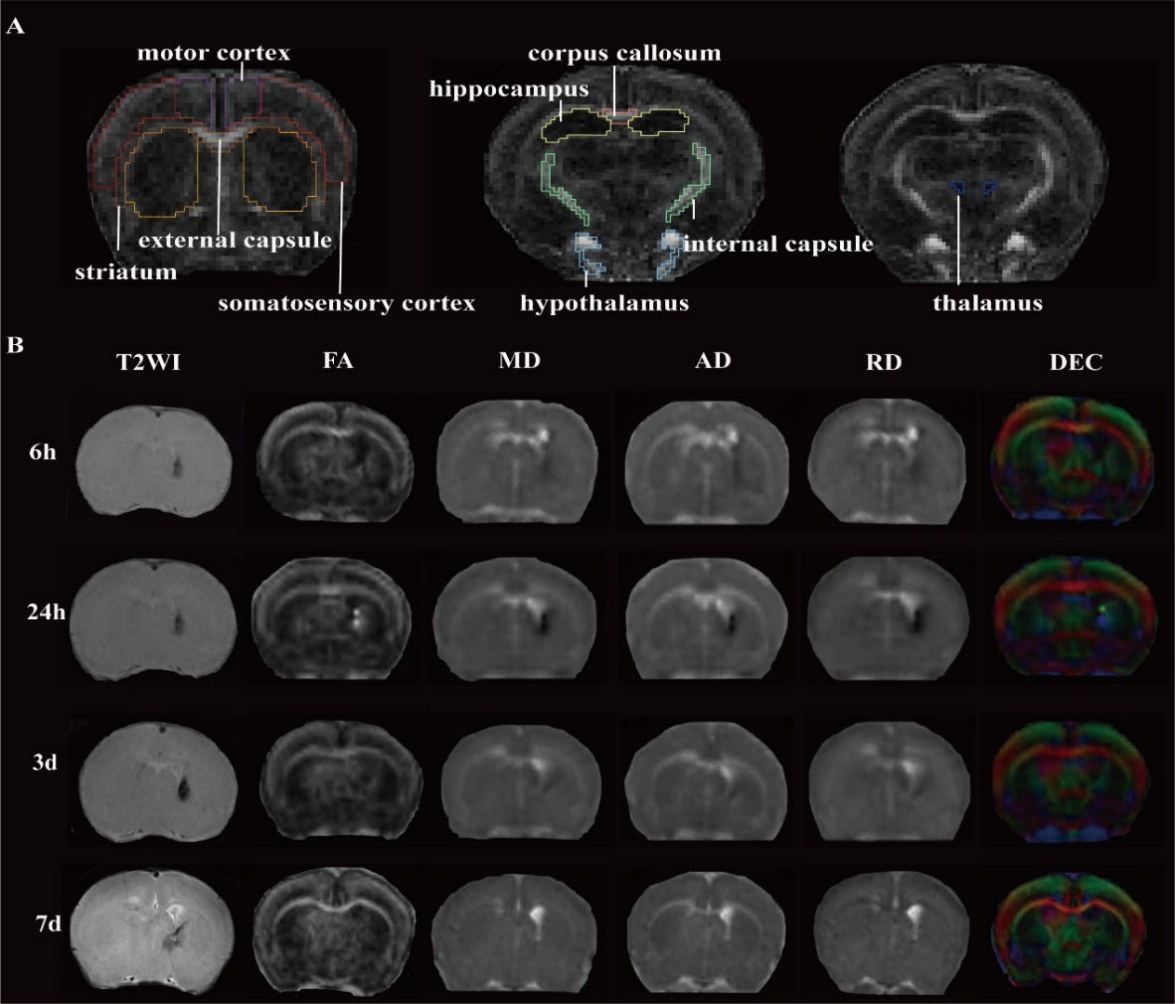
Description of the regions of interest (ROIs) and typical maps obtained with MRI protocol. (**A**) Representative images of ROIs, including the bilateral motor cortex, somatosensory cortex, striatum, corpus callosum, hippocampus, internal capsule, hypothalamus and thalamus. (**B**) Anatomical T2 weighted image (T2WI), fractional anisotropy (FA), mean diffusivity (MD), axial diffusion (AD), radial diffusion (RD) and directionally encoded colormap (DEC) images of GMH-IVH rat brain at 6 h, 24 h, 3d and 7d.

All the animals were alive 7 days after modeling, lesion in the GMH-IVH group was located in striatum which close to the germinal matrix. Figure 1A shows a manually sketched regions of interest (ROIs), which is consistent with previous research^20^. Figure 1B shows T2WI, FA, MD, AD, RD and directionally encoded colormap (DEC) maps at four time points after GMH-IVH. Compared to sham group, T2WI maps in the lesion area showed relatively uniform hypointense at 6 h, 24 h and 3 days after GMH-IVH. While, at 7 days, the lesion area showed uneven signals and spread to lateral ventricle.

We first assessed the alterations of temporal DTI parameters in striatum- lesion area.

The results of statistical analysis are summarized in Table 1, showing time effect, GMH-IVH effect and interactions (time * GMH-IVH). FA and MD values showed significant time effects, GMH-IVH effects and time * GMH-IVH interaction effects.

**Fig. 2:**
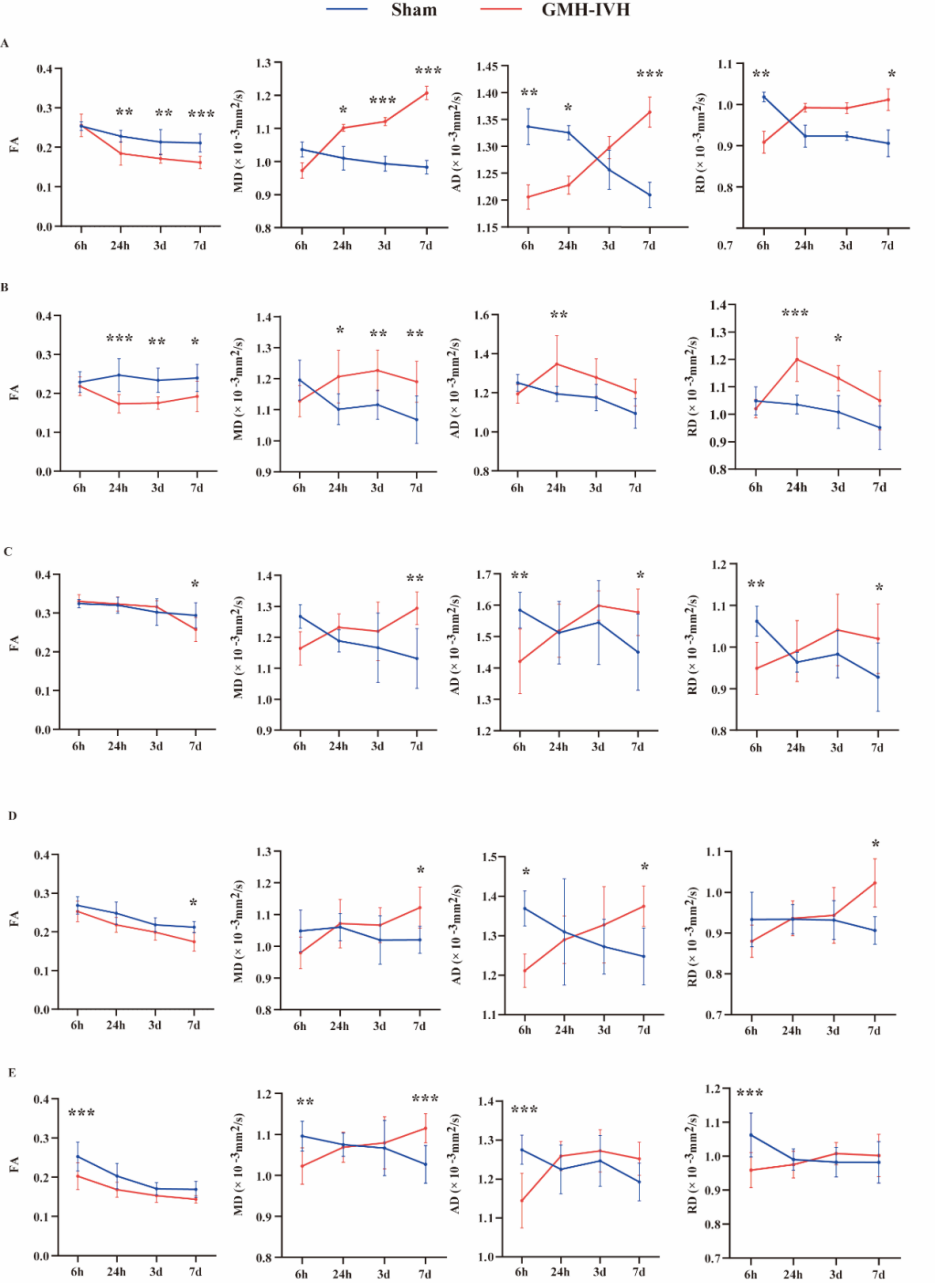
Time dependent FA, MD, AD and RD changes after GMH-IVH or sham group in selected ROIs. Graph shows FA, MD, AD and RD at the level of striatum (**A**), hippocampus (**B**), external capsule (**C**), motor cortex (**D**) and thalamus (**E**). * *p* < 0.05, ** *p* < 0.01, *** *p* < 0.001.

AD and RD values showed significant time * GMH-IVH interaction effects. The results showed that FA in striatum of GMH-IVH group decreased significantly in acute phase (at 24 h) and subacute phase (at 3 days and 7 days). On the contrary, MD values increased significantly. Meanwhile, AD and RD values were significantly lower in GMH-IVH group than sham group at the very early phase (6 h). The values increased with time and were significantly higher in GMH-IVH group than sham group at subacute phase (7 days). We further evaluate alterations of DTI parameters in other brain regions, hippocampus, thalamus, external capsule and motor cortex also showed significant differences between GMH-IVH and sham group, as shown in Fig. 2. The results showed that FA values in striatum and hippocampus of GMH-IVH group decreased significantly in acute phase (at 24 h). Oppositely, MD and AD values in striatum and hippocampus showed increased significantly in acute phase. The results also showed that FA values in external capsule and motor cortex of GMH-IVH group decreased significantly in subacute phase (at 7 days). MD values in these two brain regions were also shown to increase significantly in the subacute phase. In addition, the FA value in thalamus were significantly lower in GMH-IVH group than sham group at the very early phase (6 h). Additionally, results of statistical analysis were concluded in Supplementary Table 2. Other regions including internal capsule, hypothalamus, corpus callosum and somatosensory cortex showed no significant differences in DTI parameters between two groups at different time points. The interclass correlation coefficients (ICCs) was used to evaluate the consistency test between two observers. The results revealed that the ICC between the two observers was 0.953. The intra-observer consistency for observer 1 and observer 2 was 0.924 and 0.992, respectively.

**Table 1:** Repeated measurement ANOVA of DTI parameters in striatum after GMH-IVH injury.

ROI	ANOVA	DTI parameters			
		FA	MD	AD	RD
Striatum	Time effect	F _(1,28)_ = 36.06 *p* < 0.001***	F _(1,28)_ = 39.92 *p* < 0.001***	F _(1,28)_ = 0.208 *p* = 0.652	F _(1,28)_ = 4.367 *p* = 0.0458
	GMH-IVH effect	F _(1,28)_ = 30.11 *p* < 0.001***	F _(1,28)_ = 5.19 *p* = 0.0056**	F _(1,28)_ = 0.131 *p* = 0.941	F _(1,28)_ = 0.039 *p* = 0.989
	Time * GMH-IVH	F _(3,28)_ = 4.581 *p* = 0.0099**	F _(3,28)_ = 15.83 *p* < 0.001***	F _(3,28)_ = 12.95 *p* < 0.001***	F _(3,28)_ = 9.285 *p* = 0.0002***

Notes: FA, fractional anisotropy; MD, mean diffusion; AD, axial diffusion; RD, radial diffusion. * *p* < 0.05; ** *p* < 0.01; *** *p* < 0.001.

### Early development evaluation

**Fig. 3:**
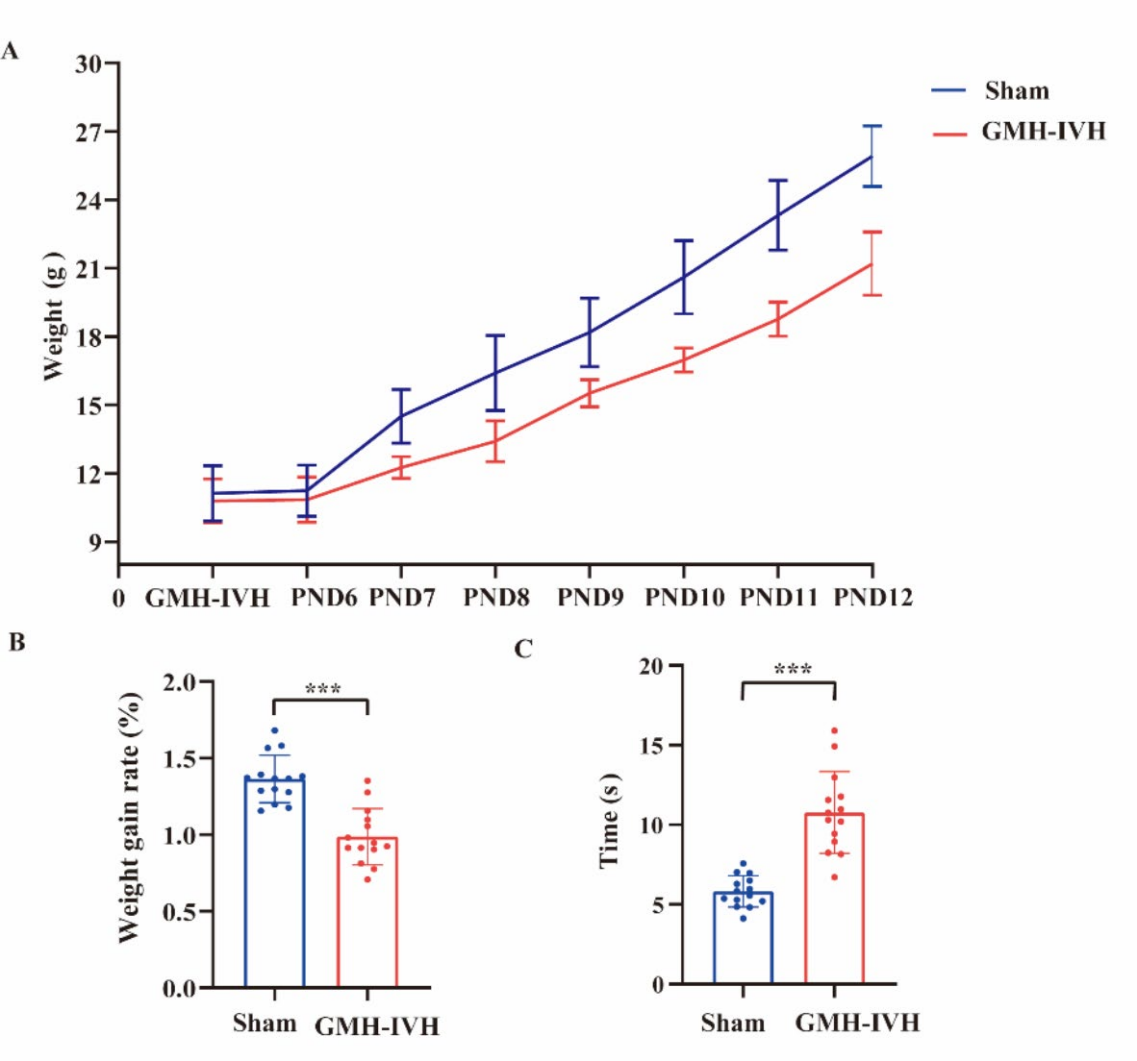
Early neurodevelopmental function evaluation following GMH-IVH. (**A**) Weight gain over time. (**B**) Weight gain rate and (**C**) negative geotaxis assessment of sham (*n* = 14) and GMH-IVH (*n* = 14) animals. ***p < 0.001.

In order to investigate the development of rat pups during early life, we compared body weight and negative geotaxis test between GMH-IVH and sham rat pups. Two indexes including raw weight and weight gain rate were used to analyze the weight changes between two groups. Line chart results of body weight changes showed that compared with rat pups in sham group, in PND 11 and PND12 GMH-IVH group, body weight was significantly decreased (Fig. 3A). In PND12, weight gain rate was significantly decreased in GMH-IVH groups compared to sham groups (Fig. 3B). The negative geotaxis test was used to evaluate motor coordination and vestibular reflexes in neonatal rats. In PND12, rotation time increased significantly in GMH-IVH group (Fig. 3C), which means the vestibular and motor coordination of GMH-IVH rat pups were decreased.

### Evaluation of neuropathological brain injury

**Fig. 4:**
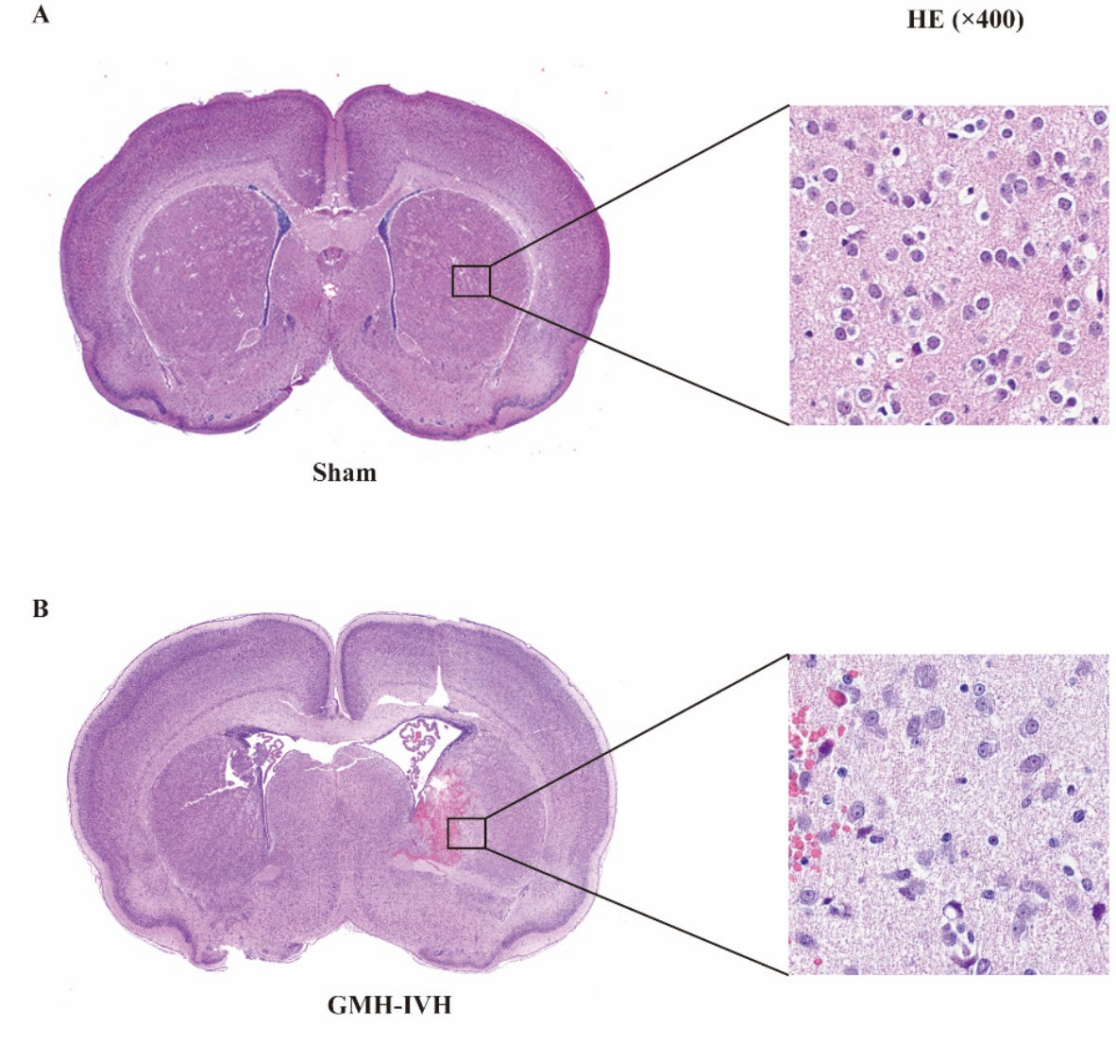
Representative picture displaying the coronal brain section of histological staining at 24 h after GMH-IVH (× 400).

H&E staining were performed on the brain sections of the Sham and GMH-IVH groups at 24 h (Fig. 4). H&E staining showed that cells in sham group were round and neatly arranged. In GMH-IVH group, cell arrangement was disordered, the cell space was enlarged, the cells were swollen, and pathological changes like “vacuole” appeared.

### Long-term neurofunctional outcomes following GMH-IVH rat pups

To further investigate the long-term neurological injury effects of GMH-IVH, we conducted two batches of behavior test, first in PND 35–42, and the second in PND 65–72. The rotarod test, open field test, Y-maze, and CatWalk system tests were included to evaluate sensory and cognitive function.

**Fig. 5:**
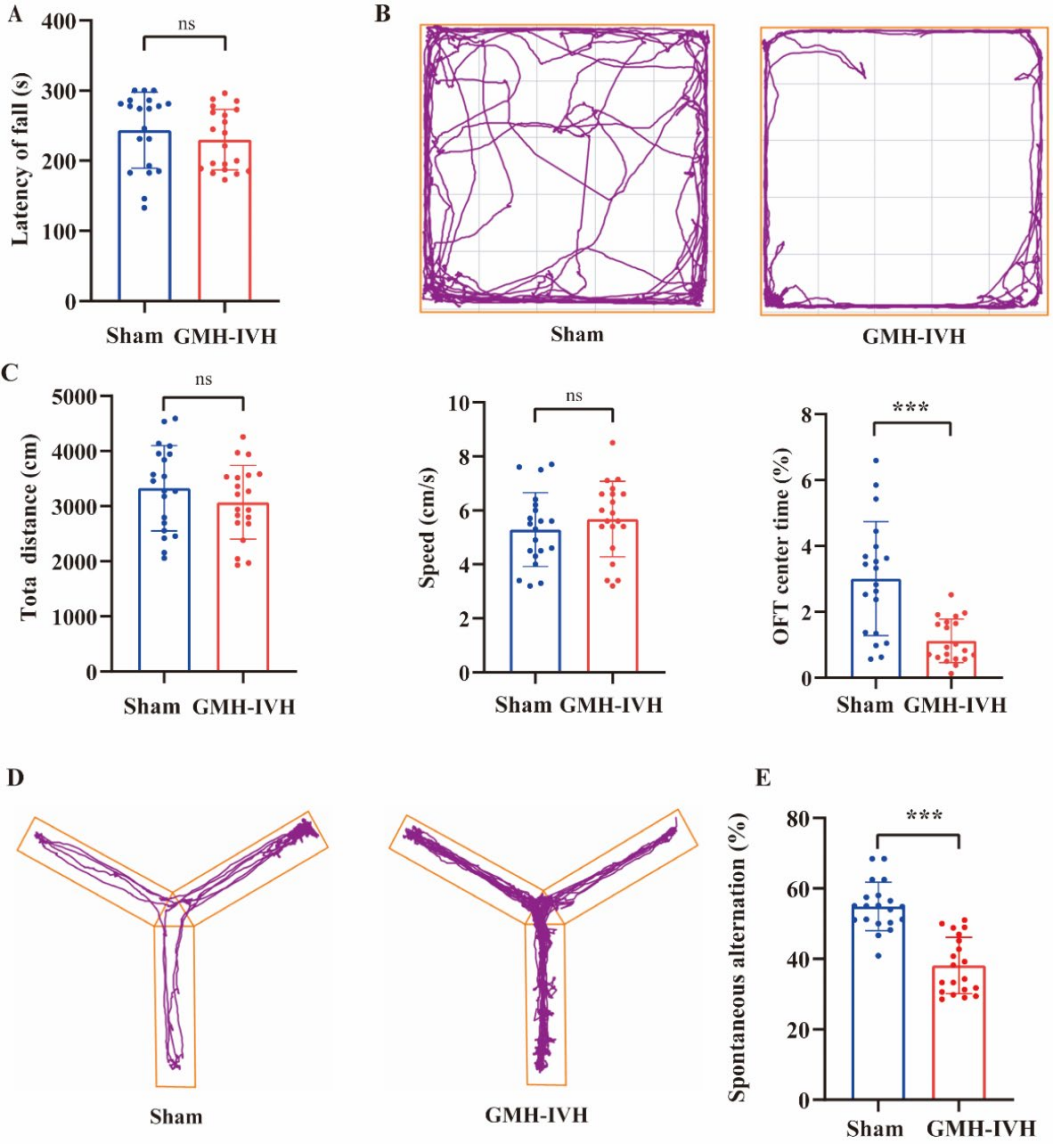
Evaluation of long-term neurofunctional outcomes following GMH-IVH rat pups. (**A**) Latency of falls in rotarod test. (**B**) Representative traces of sham and GMH-IVH rat movement during the open filed test. (**C**) Graph shows the total distance, mean speed and center time percentage in the open filed test. (**D**) Representative traces of sham and GMH-IVH rat movement during the Y maze. (**E**) Spontaneous alternation. GMH-IVH rats (*n* = 20), sham rats (*n* = 20). ***p < 0.001.

In the rotarod test (Fig. 5A), the latency of fall showed no significant difference between two groups. To explore the effect of GMH-IVH on anxiety, we examined anxiety-like behavior through open field test (Fig. 5B). There were no differences in total distance (t = 1.614, *p* = 0.1196) and speed (t = 0.984, *p* = 0.3348) between GMH-IVH and sham rat pups (Fig. 5C). However, percentage of time in center showed significant difference (t = 4.560, *p* < 0.001). The result showed GMH-IVH could affect anxiety-like behavior. Y-maze (Fig. 5D) was used to evaluate spontaneous activity. GMH-IVH group showed decreased spontaneous alteration compared with sham group (Fig. 5E).

**Fig. 6:**
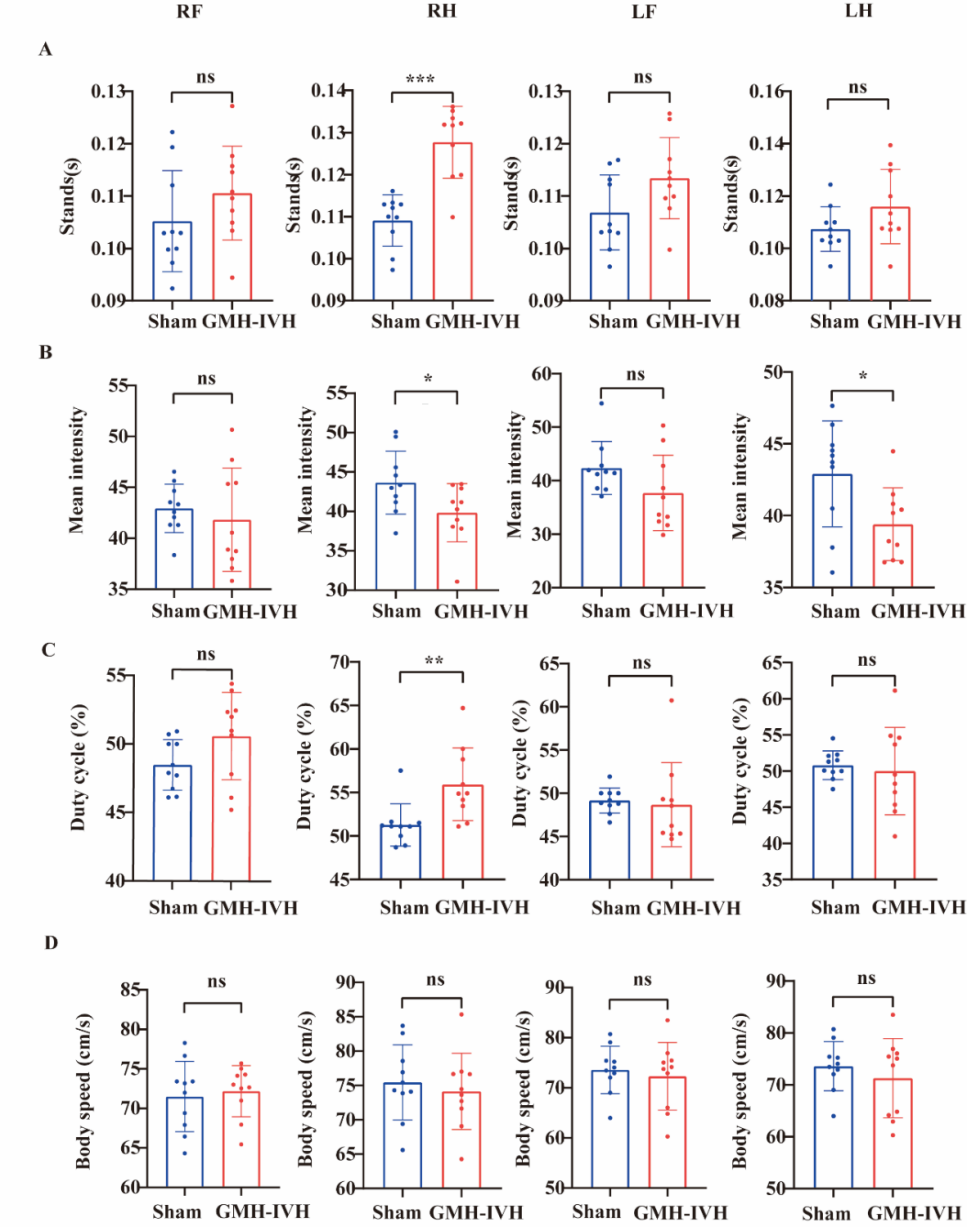
GMH-IVH induced motor dysfunction in gait analysis. (**A**) Stands. (**B**) Mean intensity. (**C**) Duty cycle (%) and (**D**) body speed. Data are displayed as a bar chart of mean values (*n* = 10/group), *p < 0.05, ** *p* < 0.01, ***p < 0.001. RF: right front limb; RH: right hind limb; LF: left front limb; LH: left hind limb.

Next, we further evaluated whether GMH-IVH causes motor dysfunction by CatWalk XT system. Gait analysis by CatWalk XT showed that the stands of right hind (RH) paw were highly increased in GMH-IVH group compared to sham group (t = 5.599, *p* < 0.001). Other paws, including right front (RF), left front (LF) and left hind (LH) showed no significant difference of stands between two groups (Fig. 6A). The mean intensity of footprints of right hind (t = 2.211, *p* = 0.0402) and left hind (t = 2.477, *p* = 0.02334) were significantly decreased in GMH-IVH group (Fig. 6B). In order to investigate whether there was any lateralization, we further calculated the duty cycle among four paws. Results showed that the duty cycle of right hind increased significantly in GMH-IVH group compared to sham group. Other paws showed no difference between two groups (Fig. 6C). Additionally, speed (Fig. 6D) or other parameters displayed no significant changes between GMH-IVH group and sham group.

**Fig. 7:**
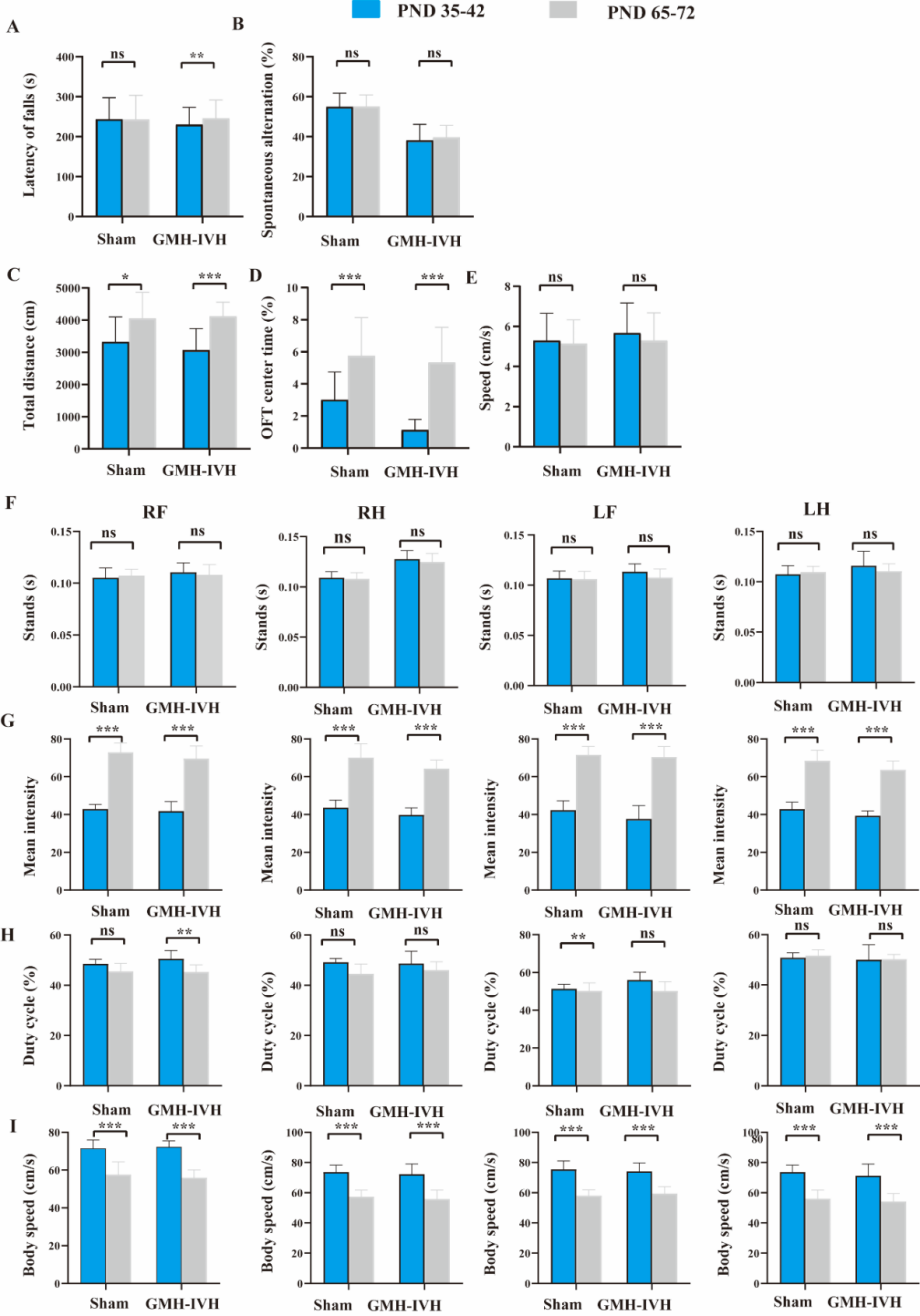
Comparison between the two behavioral test batches both in sham and GMH-IVH group. * *p* < 0.05, ** *p* < 0.01, *** *p* < 0.001.

In addition, we examined the second batch of neurobehavior tests in PND65-72. And only rotarod test and CatWalk system tests showed significant differences, other tests did not show any positive results (supplementary Fig. 1). To better evaluate the developmental changes of the two groups, we compared the two behavioral test batches (Fig. 7).

### Correlation analysis of DTI parameters and long-term neurofunctional outcomes

Considering the consequences of MRI, we focused on conducted a correlation analysis of DTI parameter values at 24 h in striatum, hippocampus, external capsule, motor cortex and thalamus with long-term behavior outcomes, as shown in Table 2. The results showed that no DTI parameters were related to OFT center time, spontaneous alternation or LF mean intensity. The FA values in the hippocampus and motor cortex negatively correlated with RH stands (*r* = -0.733, *p* < 0.001, and *r* = -0.606, *p* = 0.013, respectively). The AD values in striatum also negatively correlated with RH stands (*r* = -0.694, *p* = 0.003). However, the MD, AD and RD values in the hippocampus positively correlated with RH stands (*r* = 0.527, *p* = 0.036, *r* = 0.645, *p* = 0.007, and *r* = 0.764, *p* < 0.001, respectively). Only the RD value in thalamus positively correlated with RH mean intensity (*r* = 0.840, *p* < 0.001). The FA and AD values in striatum negatively correlated with RH duty cycle (*r* = -0.634, *p* = 0.008, and *r* = − 0.545, *p* = 0.029, respectively). In addition, the RD value in hippocampus and MD value in external capsule positively correlated with RH duty cycle (*r* = 0.579, *p* = 0.019, and *r* = 0.670, *p* = 0.005, respectively).

**Table 2:** The correlation analysis of DTI tractography data (FA, MD, AD and RD) and long-term neurofunctional outcomes.

		OFT Center time (%)	Spontaneous alternation (%)	RH Stands (s)	RH Mean intensity	LH Mean intensity	RH Duty Cyte(%)
Striatum	FA	*r* = 0.089 *p* = 0.743	*r* = -0.048 *p* = 0.859	*r* = -0.462 *p* = 0.071	*r* = 0.062 *p* = 0.820	*r* = -0.006 *p* = 0.983	*r* = -0.634 *p* = 0.008**
	MD	*r* = 0.223 *p* = 0.407	*r* = 0.052 *p* = 0.849	*r* = 0.140 *p* = 0.605	*r* = 0.205 *p* = 0.447	*r* = 0.335 *p* = 0.204	*r* = 0.431 *p* = 0.096
	AD	*r* = 0.010 *p* = 0.971	*r* = 0.041 *p* = 0.881	*r* = -0.694 *p* = 0.003**	*r* = 0.374 *p* = 0.153	*r* = -0.284 *p* = 0.286	*r* = -0.545 *p* = 0.029*
	RD	*r* = 0.087 *p* = 0.750	*r* = 0.169 *p* = 0.532	*r* = 0.328 *p* = 0.214	*r* = 0.337 *p* = 0.201	*r* = -0.002 *p* = 0.993	*r* = 0.130 *p* = 0.631
Hippocampus	FA	*r* = 0.236 *p* = 0.379	*r* = 0.128 *p* = 0.637	*r* = -0.733 *p* < 0.001**	*r* = 0.065 *p* = 0.810	*r* = -0.083 *p* = 0.760	*r* = -0.436 *p* = 0.091
	MD	*r* = -0.061 *p* = 0.822	*r* = -0.170 *p* = 0.529	*r* = 0.527 *p* = 0.036*	*r* = -0.078 *p* = 0.775	*r* = 0.008 *p* = 0.975	*r* = 0.466 *p* = 0.069
	AD	*r* = -0.145 *p* = 0.592	*r* = 0.039 *p* = 0.885	*r* = 0.645 *p* = 0.007**	*r* = -0.079 *p* = 0.771	*r* = -0.128 *p* = 0.637	*r* = 0.306 *p* = 0.250
	RD	*r* = 0.048 *p* = 0.861	*r* = -0.073 *p* = 0.789	*r* = 0.764 *p* < 0.001**	*r* = -0.183 *p* = 0.499	*r* = 0.088 *p* = 0.745	*r* = 0.579 *p* = 0.019*
External capsule	FA	*r* = -0.571 *p* = 0.021*	*r* = -0.044 *p* = 0.872	*r* = -0.016 *p* = 0.954	*r* = -0.205 *p* = 0.447	*r* = -0.142 *p* = 0.600	*r* = -0.057 *p* = 0.834
	MD	*r* = -0.063 *p* = 0.817	*r* = -0.073 *p* = 0.789	*r* = 0.363 *p* = 0.167	*r* = -0.031 *p* = 0.910	*r* = 0.106 *p* = 0.696	*r* = 0.670 *p* = 0.005**
	AD	*r* = -0.166 *p* = 0.538	*r* = 0.290 *p* = 0.275	*r* = 0.054 *p* = 0.842	*r* = -0.038 *p* = 0.888	*r* = -0.390 *p* = 0.135	*r* = -0.004 *p* = 0.987
	RD	*r* = -0.171 *p* = 0.527	*r* = -0.304 *p* = 0.252	*r* = 0.404 *p* = 0.120	*r* = -0.277 *p* = 0.299	*r* = -0.400 *p* = 0.125	*r* = 0.315 *p* = 0.234
Motor cortex	FA	*r* = -0.430 *p* = 0.097	*r* = -0.003 *p* = 0.991	*r* = -0.606 *p* = 0.013*	*r* = 0.119 *p* = 0.660	*r* = 0.177 *p* = 0.511	*r* = -0.276 *p* = 0.300
	MD	*r* = -0.083 *p* = 0.759	*r* = 0.237 *p* = 0.377	*r* = -0.041 *p* = 0.879	*r* = 0.128 *p* = 0.636	*r* = -0.357 *p* = 0.175	*r* = -0.046 *p* = 0.867
	AD	*r* = -0.050 *p* = 0.854	*r* = 0.095 *p* = 0.725	*r* = -0.209 *p* = 0.437	*r* = 0.440 *p* = 0.088	*r* = 0.117 *p* = 0.667	*r* = -0.097 *p* = 0.721
	RD	*r* = 0.006 *p* = 0.981	*r* = -0.146 *p* = 0.589	*r* = -0.027 *p* = 0.921	*r* = 0.152 *p* = 0.574	*r* = -0.172 *p* = 0.523	*r* = -0.037 *p* = 0.890
Thalamus	FA	*r* = 0.274 *p* = 0.305	*r* = 0.241 *p* = 0.369	*r* = -0.410 *p* = 0.114	*r* = 0.214 *p* = 0.426	*r* = -0.024 *p* = 0.930	*r* = -0.386 *p* = 0.140
	MD	*r* = 0.054 *p* = 0.843	*r* = 0.123 *p* = 0.651	*r* = -0.007 *p* = 0.981	*r* = 0.338 *p* = 0.201	*r* = 0.167 *p* = 0.536	*r* = -0.091 *p* = 0.737
	AD	*r* = 0.320 *p* = 0.227	*r* = -0.063 *p* = 0.816	*r* = -0.036 *p* = 0.895	*r* = 0.483 *p* = 0.058	*r* = -0.055 *p* = 0.838	*r* = 0.035 *p* = 0.898
	RD	*r* = -0.075 *p* = 0.781	*r* = 0.105 *p* = 0.700	*r* = -0.592 *p* = 0.016*	*r* = 0.840 *p* < 0.001***	*r* = -0.136 *p* = 0.617	*r* = -0.479 *p* = 0.061

Notes: FA, fractional anisotropy; MD, mean diffusion; AD, axial diffusion; RD, radial diffusion. * *p* < 0.05; ** *p* < 0.01; *** *p* < 0.001.

### Brain transcriptome analysis reveals key gene expression and involved pathways

**Fig. 8:**
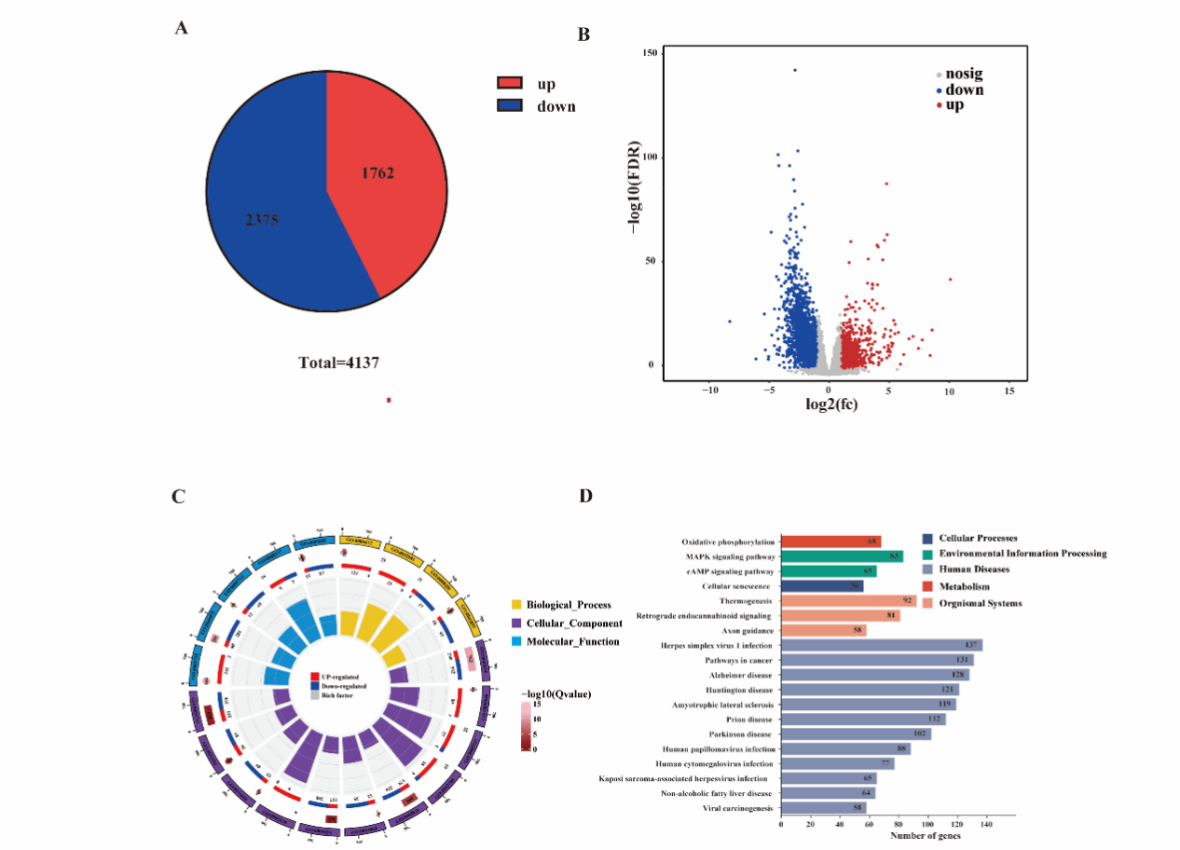
Transcriptome analysis after 24 h of GMH-IVH. Differentially expressed gene statistics (**A**) and the volcano plot of sham vs. GMH-IVH (**B**). (**C**) GO enrichment circle map. (**D**) Top 20 KEGG enrichment pathway annotations.

To further explore the potential molecular mechanisms involved in the brain injury induced by GMH-IVH, we performed RNA-seq of ipsilateral cerebral hemisphere collected at 24 h after saline or collagenase injection. As shown in this figure (Fig. 8A), 1762 genes were significantly upregulated, and 2135 genes were significantly down regulated. The volcano plot (Fig. 8B) depicted the genes expression patterns including the number of down-regulated, up-regulated and genes with no significant differences. To confirm the reliability of this data, the correlation between the samples were performed (Supplementary Fig. 2). Among the top 20 most upregulated genes (Supplementary Table 3), some were encoding for hemoglobin components (Hba-a1 and Hba-a2). Figure 8C shows the GO enrichment circle map of DEGs. As it can be seen from the figure, a large number of genes involved in cellular functions, followed by molecular functions, while fewer genes are involved in biological processes. In order to further explore the changes involved in biological pathways, KEGG enrichment analysis were conducted. And top 20 pathways can be seen on Fig. 8D, including five classes: human diseases, organismal systems, environmental information processing, cellular processes and metabolism.

## Discussion

GMH-IVH is a common form of intracranial hemorrhage in preterm neonates. This hemorrhage originates from subependymal germinal matrix and can destroy the endymal lining and spread to the lateral ventricle. In this study, DTI technique was used to monitor the brain tissue alterations over time. Results showed that DTI parameters play an important role in early identification GMH-IVH brain injury.

T2WI MRI can detect the size of cerebral hemorrhage. In this study, we first performed T2WI scanning between GMH-IVH group and sham group. The results showed that the volume of cerebral hemorrhage changed over time and eventually infiltrated the lateral ventricle. DTI measures and records signals of the movement of water molecules in small imaging volumes voxels. FA values are related to the brain microscopic changes affected by GMH-IVH^17^. Based on the application of DTI imaging technology in other animal models^21–23^, we chose to include four basic parameters, including AD, RD, MD, and FA as the temporal and spatial tracking of brain injury in GMH-IVH, and compared to sham group. Results showed that FA values in striatum and hippocampus decreased significantly at 24 h in GMH-IVH, while AD, RD and MD increased significantly. Previous studies have shown a lower FA indicates lower anisotropic diffusion and lower microstructure integrity. At subacute phase (7 days), FA values in external capsule and motor cortex showed significantly decreased in GMH-IVH group. And MD values showed the opposite result. These are likely to explain the differences in early neurobehavioral outcomes at 7 days (PND12) after GMH-ICH. These results also support the use of DTI imaging as an early noninvasive method to assess changes in brain microstructure in GMH-IVH animal model. In addition, AD and RD may provide additional information on the underlying mechanisms of white matter integrity. At PND35, FA values measured in hippocampus and thalamus in GMH-IVH group still lower than sham group. These results were similar to X. Zhang et al. research. All of these results mean that GMH-IVH can induce damage to white matter. Vernooij et al.^24^ also showed that white matter injury was associated with increased MD and decreased FA.

HE staining allows for the clear observation of the morphology and structure of tissues and cells, providing crucial evidence for the diagnosis of diseases. Hu et al.^25^ noticed that the glomerulosclerosis and tubular damage could be the underlying mechanisms for decreased FA values in diabetic rat kidneys. In this study, we observed significant pathological alterations in the GMH-IVH group at 24 h, characterized by disorganized brain tissue cellular arrangement, enlarged intercellular spaces, and cellular swelling. These pathological manifestations were predominantly localized in the perilesional area surrounding the hemorrhage site, specifically within the striatum. This observation aligns with our previous findings demonstrating a marked reduction in fractional anisotropy (FA) values within the striatum of the GMH-IVH group during the acute phase at 24 h. Based on these correlative findings, we hypothesized that the observed decrease in FA values may be attributed to the disruption of tissue architecture and cellular edema in the GMH-IVH model. In preterm neonates^9,26^, mild low-level GMH-IVH can induce impaired neurological function. In GMH-IVH model, we also performed neurobehavioral assessments. In the early stages, our results showed that weight gain rate in the GMH-IVH group was significantly lower than sham group. Rat pups in GMH-IVH group took more time in the negative geotaxis test. These results were consistent with Jinnai et al. results^18^. In order to better evaluate the motor and cognitive function of the GMH-IVH rat pups, we conducted long-term behavioral experiments at two time points. First in PND 35–44, our results showed that rat pups in GMH-IVH group took more time in center regions compared to sham group in OFT. This means that rat pups injected with collagenase experienced anxiety-like behavior. However, X. Zhang et al.^17^ showed that they did not observe the significant difference in OFT between GMH group and sham group. In addition, similar results were found in the elevated plus maze. We suspected that the reason for the discrepancy with our results is mainly due to the difference in test time. In X. Zhang et al. study, they performed OFT at PND 59, while our study conducted at PND 35. Rodents have strong neurobehavioral resilience after brain injury, and may return to neurobehavioral abnormalities if behavioral tests are not performed shortly after brain injury. Our results suggest that 30 days after GMH-IVH model has better evaluatiove significance than other long-term behavior time points. To further support our suggestion, we also conducted tests at the second time point (PND 65–72). Simliar to X. Zhang et al.’s results, there were no significantly positive results in other neurobehavioral experiments except gait analysis experiments. Additionally, to better evaluate the developmental changes, we compared the two behavioral test batches in both groups. The results showed that the motor function and anxiety-like behavior of GMH-IVH mice slightly improved over time. For example, in the rotarod test, the latency of fall in GMH-IVH (PND 65–72) is significantly higher than GMH-IVH (PND 35–42). In OFT, both the sham group and the GMH-IVH group showed that the percentage of time in center increased significantly. In gait analysis, we looked some changes between the two behavioral test batches. Furthermore, the significant differenes in gait analysis experiments between two groups at PND 65–72 were also noted. Consistence with the results of other studies, injection of collagenase induce motor dysfunction in rats, especially for the paw of right hind.

In other animal models, researchers analyzed correlations between FA values in specific brain regions and behaviors. Liu, Qian et al.^27^ showed that FA values in corpus colusum and external capsulr positively correlated with alternations in Y maze test (*r* = 0.473, *p* < 0.05, and *r* = 0.64, *p* < 0.01, respectively) in chronic cerebral hypoperfusion model with bilateral cartoid artery stenosis. In this study, we also conducted correlations between DTI parameters and long-term behavioral outcome. We noticed that there was no DTI parameter were related to spontaneous alternation in Y maze test. But, other neurofunctional behaviors including RH stands, RH mean intensity and RH duty cycle were related to DTI parameters at acuter phase. Transcriptome analysis can unravel the underlying molecular mechanisms. Based on the DTI measurements, we focused on evaluating transcriptomics 24 h after GMH-IVH. In our results, the most significant upregulated DEGs in top 20 upregulated genes were Hba-al and Hba-a2, which coding for the hemoglobin component. These genes were similar to the previous research^28^. However, the number of DEGs in this study much more than Song et al.’s study. The possible reason is that the study of Song et al. focused on the transcriptomics of the right temporal lobe, while in our study, we chose the brain hemisphere of the injured side for sequencing analysis. Meanwhile, our results also revealed the top 20 possible KEGG-related pathways, which laid the foundation for further research on the mechanism of GMH-IVH.

This study has some advantages. Firstly, DTI parameters alterations in a model of GMH-IVH in preterm rat pups were evaluated for the first time. Emphasizing the ability of DTI technology to noninvasively detect brain microstructural changes at an early stage. Additionally, we evaluated early and long-term behavioral studies to lay the foundation for subsequent mechanistic studies. We also used the transcriptome study to analyze the possible molecular mechanisms after 24 h of GMH-IVH, which provided the direction for our further research. Our study still has some limitations. Firstly, the sample size of this study is relatively small and the effect of sex not explored. In other model mice or rats, many researchers have noticed gender issues^20,29^. Large-sample studies are need to further replicate and the effect of sex should be included. Secondly, although DTI is an effective tool to track and monitor brain changes at the microstructural level, it is based on the assumption of tissue isotropy. However, previous studies have taken into account the presence of anisotropy in tissues or effect of cerebral blood flow, using more advanced MRI techniques such as diffusion kurtosis imaging^30–32^ and arterial spin labelling^33^. In the follow-up experiments, we could consider adding more MRI techniques. Finally, our research focused on the variation of DTI parameter in GMH-IVH and sham groups. The correlations between long-term behaviors and DTI parameters were also analyzed. And we did transcriptomic analysis to try uncovering the mechanisms involved in GMH disease. However, the pathogenesis is complex in GMH and it’s worth studying deeply. Subsequent researches need to further investigate the correlation between MRI parameters and possible pathogenesis, including neuroinflammation, oxidative stress and so on.

## Conclusion

Our study showed that DTI could be an effective and sensitive technique for evaluating brain injury changes in neonatal rat GMH model. FA, MD, AD and RD might uncover injury brain microstructure changes at early development, which could serve as noninvasive and reliable markers in GMH model.

## Electronic supplementary material

Below is the link to the electronic supplementary material.

Supplementary Material 1

## Data Availability

The datasets used and/or analyzed during the current study are available from the corresponding author on reasonable request. A submission to the journal implies that materials described in the manuscript, including all relevant raw data, will be freely available to any researcher wishing to use them for non-commercial purposes, without breaching participant confidentiality.
