# Supplementary material for: Preliminary feasibility study on DTI to assess the early brain injury in germinal matrix-intraventricular hemorrhage rats
Source: Sci Rep. 2025 Mar 21;15:9839. doi: 10.1038/s41598-025-94934-x (PMC11928497; doi:10.1038/s41598-025-94934-x)
Supplement: Supplementary file 1 — Supplementary Material 1 [file 41598_2025_94934_MOESM1_ESM.docx]

**Supplementary Table 1**

The allocation of animals in this study.

|  | Sham | GMH-IVH |
| --- | --- | --- |
| MRI measurements | 11 | 11 |
| Evaluation of early development | 14 | 14 |
| Rotarod test  Open field test  Y-maze | 20 | 20 |
| Gait analysis | 10 | 10 |
| H&E staining | 5 | 5 |
| RNA-sequencing | 5 | 5 |

**Supplementary Table 2**

| ROI | ANOVA | DTI parameters | | | |
| --- | --- | --- | --- | --- | --- |
|  |  | FA | MD | AD | RD |
| Hippocampus | Time effect | F _(1,28)_ = 38.41  *p* < 0.001*** | F _(1,28)_ = 17.53  *p* < 0.001*** | F _(1,28)_ = 12.8  *p* = 0.0013** | F _(1,28)_ = 23.11  *p* < 0.001*** |
|  | GMH-IVH effect | F _(1,28)_ = 1.233  *p* = 0.316 | F _(1,28)_ = 1.258  *p* = 0.307*** | F _(1,28)_ = 7.594  *p* < 0.001*** | F _(1,28)_ = 12.42  *p* < 0.001*** |
|  | Time * GMH-IVH | F _(3,28)_ = 3.066  *p* = 0.044* | F _(3,28)_ = 7.86  *p* < 0.001*** | F _(3,28)_ = 4.607  *p* = 0.0096** | F _(3,28)_ = 5.001  *p* < 0.0067** |
| External capsule | Time effect | F _(1,28)_ = 0.905  *p* = 0.349 | F _(1,28)_ = 3.353  *p* = 0.078 | F _(1,28)_ = 0.072  *p* = 0.791 | F _(1,28)_ = 0.939  *p* = 0.341 |
|  | GMH-IVH effect | F _(1,28)_ = 10.68  *p* < 0.001*** | F _(1,28)_ = 0.565  *p* = 0.643 | F _(1,28)_ = 1.541  *p* = 0.226 | F _(1,28)_ = 1.351  *p* = 0.278 |
|  | Time * GMH-IVH | F _(3,28)_ = 5.242  *p* = 0.0054** | F _(3,28)_ = 6.552  *p* = 0.0017** | F _(3,28)_ = 7.776  *p* < 0.001*** | F _(3,28)_ = 7.398  *p* < 0.001*** |
| Motor cortex | Time effect | F _(1,28)_ = 21.08  *p* < 0.001*** | F _(1,28)_ = 2.081  *p* = 0.160 | F _(1,28)_ = 0.003  *p* = 0.953 | F _(1,28)_ = 1.894  *p* = 0.876 |
|  | GMH-IVH effect | F _(1,28)_ = 27.19  *p* < 0.001*** | F _(1,28)_ = 3.294  *p* = 0.035* | F _(1,28)_ = 0.174  *p* = 0.913 | F _(1,28)_ = 4.465  *p* = 0.011** |
|  | Time * GMH-IVH | F _(3,28)_ = 0.805  *p* = 0.5015 | F _(3,28)_ = 5.086  *p* = 0.0062** | F _(3,28)_ = 11.3  *p* < 0.001*** | F _(3,28)_ = 0.754  *p* = 0.529 |
| Thalamus | Time effect | F _(1,28)_ = 23.34  *p* < 0.001*** | F _(1,28)_ = 0.300  *p* = 0.588 | F _(1,28)_ = 0.434  *p* = 0.5155 | F _(1,28)_ = 2.497  *p* = 0.125 |
|  | GMH-IVH effect | F _(1,28)_ = 30.57  *p* < 0.001*** | F _(1,28)_ = 0.218  *p* = 0.883 | F _(1,28)_ = 0.041  *p* = 0.841 | F _(1,28)_ = 5.352  *p* = 0.0048** |
|  | Time * GMH-IVH | F _(3,28)_ = 1.125  *p* = 0.3556 | F _(3,28)_ = 12.42  *p* < 0.001*** | F _(3,28)_ = 9.627  *p* < 0.001*** | F _(3,28)_ = 6.649  *p* < 0.001*** |

Repeated measurement ANOVA of DTI parameters in ROIs after GMH-IVH injury.

Notes: **p* < 0.05, ** *p* < 0.01, ****p* < 0.001.

Notes: FA, fractional anisotropy; MD, mean diffusion; AD, axial diffusion; RD, radial diffusion. * p < 0.05; ** p < 0.01; *** p < 0.001

**Supplementary Figure 1**

GMH-IVH induced motor dysfunction in gait analysis at PND 65-72.

(A) Stands, (B) Mean intensity, (C) Duty cycle (%) and (D) body speed. Data are displayed as a bar chart of mean values (n = 12/group), **p* < 0.05, ** *p* < 0.01, ****p* < 0.001. RF: right front limb; RH: right hind limb; LF: left front limb; LH: left hind limb.


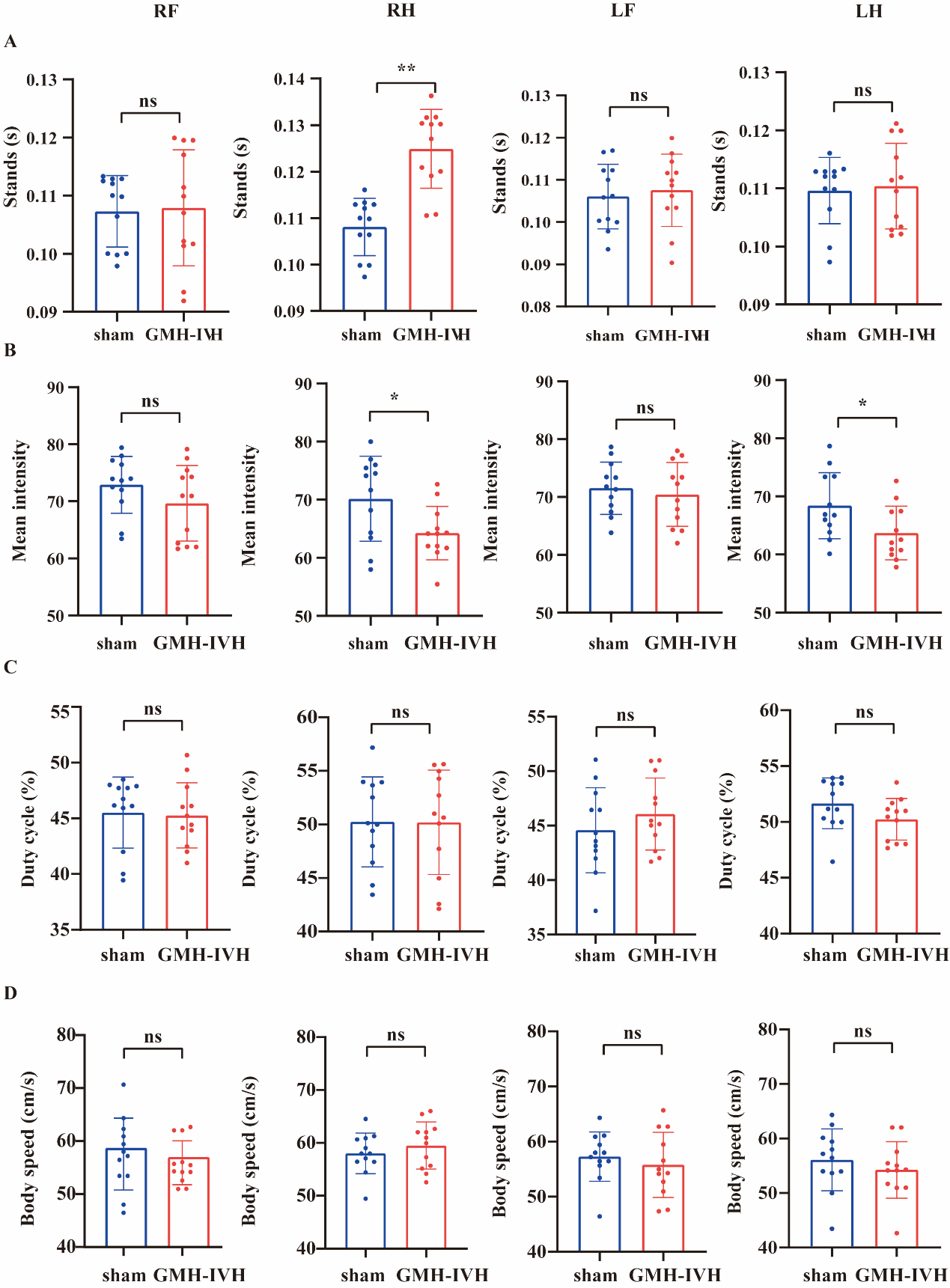


**Supplementary Figure 2**

Sample correlation heat map of Sham vs GMH-IVH. Notes: S, Sham; G, GMH-IVH.


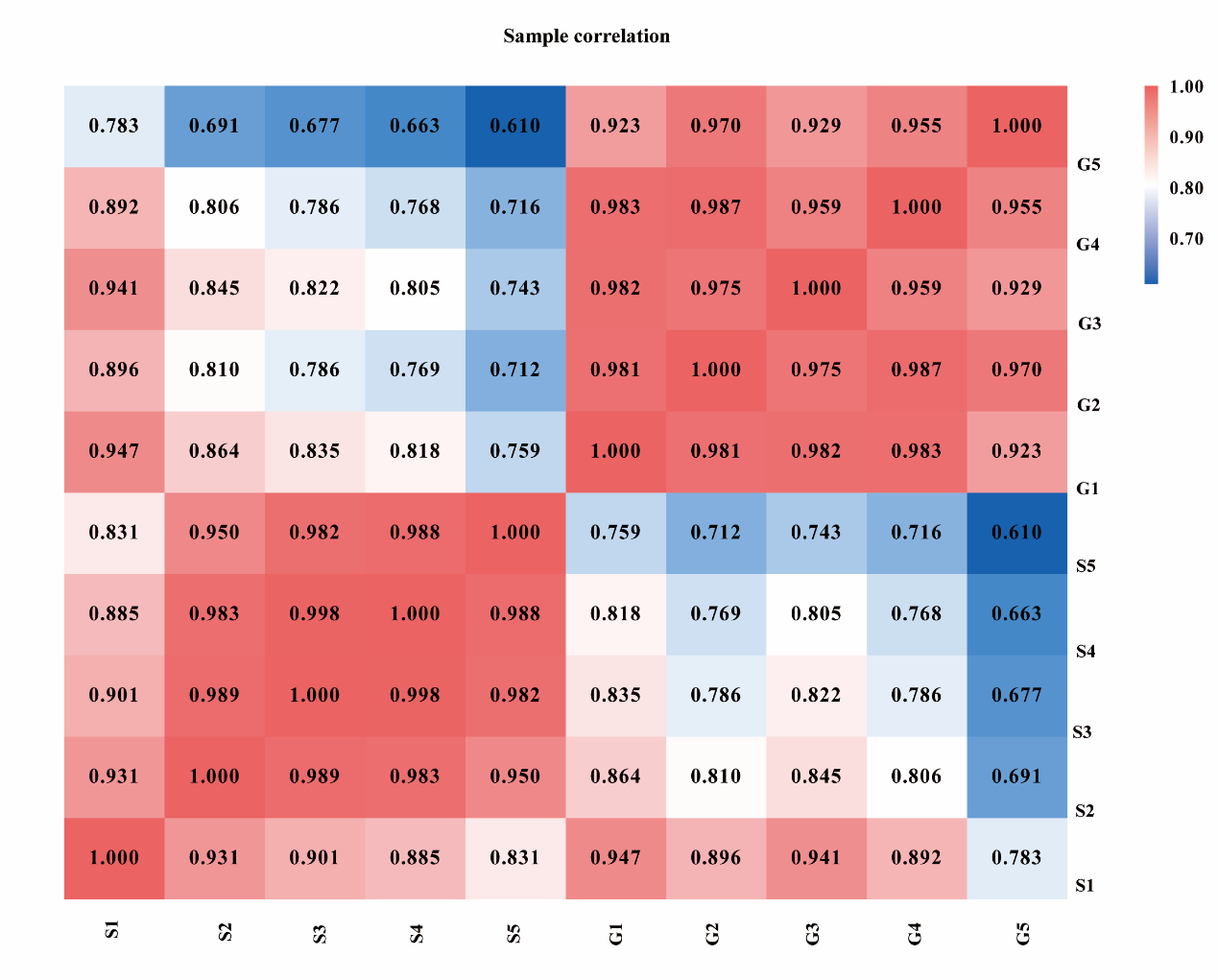


**Supplementary Table 3**

**The top 20 most up regulated DEGs in transcriptome analysis.**

| **Gene name** | **Adj. *p*-value** | **Log2FC** |
| --- | --- | --- |
| AABR07015078.1 | 1.44E-92 | 4.79 |
| AABR07063424.1 | 1.97E-65 | 4.60 |
| Anxa3 | 1.04E-64 | 1.80 |
| Ifitm3 | 3.18E-62 | 3.99 |
| Hmox1 | 2.54E-56 | 4.08 |
| AABR07015081.2 | 5.55E-56 | 3.25 |
| C1qb | 1.07E-54 | 4.47 |
| Ttr | 1.61E-46 | 1.66 |
| Timp1 | 1.05E-44 | 10.09 |
| Hba-a1 | 1.99E-44 | 3.19 |
| Hspb1 | 5.34E-44 | 3.61 |
| Alas2 | 6.96E-44 | 4.03 |
| Lgals5 | 3.24E-42 | 3.62 |
| C1qc | 3.17E-38 | 3.59 |
| Hba-a2 | 1.26E-36 | 1.45 |
| Ctsz | 1.17E-35 | 3.62 |
| Gfap | 6.41E-35 | 2.23 |
| Clic1 | 7.58E-35 | 3.31 |
| Mmp9 | 3.79E-33 | 3.53 |
| Vim | 4.07E-33 | 2.01 |
